# WGCNA-identified COL13A1 drives osteosarcoma metastasis and progression via TGF-β signaling

**DOI:** 10.1016/j.jbo.2025.100721

**Published:** 2025-11-02

**Authors:** Kang-Wen Xiao, Zhenyi Chen, Chong Zhang, Zhiqiang Yang, Liangyu Guo, Yuanlong Xie, Jun Lei, Lin Cai

**Affiliations:** a Department of Spine Surgery and Musculoskeletal Tumor, Zhongnan Hospital of Wuhan University, Wuhan, Hubei, China; b Department of Oncology, Washington University in St. Louis, St Louis, MO USA; c Department of Orthopedics, Renmin Hospital of Wuhan University, Wuhan, Hubei, China

**Keywords:** COL13A1, Osteosarcoma, Weighted Gene Co-Expression Network Analysis, TGF-β, Immunotherapy

## Abstract

Osteosarcoma (OS) is a malignant bone tumor with high incidence of metastasis. However, the molecular landscape of osteosarcoma remains incompletely understood. Weighted gene co-expression network analysis (WGCNA), differential expressed genes (DEGs), Cox regression, gene set enrichment analysis (GSEA), receiver operating characteristic curve (ROC) and survival analysis were conducted to screen potential targets and molecular mechanism for OS. Seven modules were considered to be closely related to the prognosis of OS. Subsequent immunohistochemistry (IHC), survival and ROC analysis indicated that high expression of COL13A1 was seen in OS tissue and was significantly associated with poor clinical outcome. COL13A1 expression may correlate with inhibition of M1 polarization and could serve as a predictor for immunotherapy response. Further cellular experiments showed that the expression of COL13A1 promoted the proliferation, migration and invasion. Besides, high expression of COL13A1 enhanced TGF-β signaling through β1 integrin and upregulated MMP9 and cyclin D1 expression. Finally, the low expression of COL13A1 limited the weight and lung metastasis of tumor, and reduced bone destruction in the orthotopic tumor-bearing model. COL13A1 was identified as a novel regulator of OS progression via TGF-β signaling, suggesting its potential as a therapeutic target pending further validation.

## Highlights

• Through WGCNA of the TARGET-OS cohort, COL13A1 emerged as a prognostic gene in osteosarcoma.
• COL13A1 effectively promoted osteosarcoma proliferation, metastasis, and bone destruction.
• COL13A1 enhanced TGF-β signaling through β1 integrin.
• The study confirmed the role of COL13A1 in osteosarcoma progression.

## Graphical abstract

**Figure:**
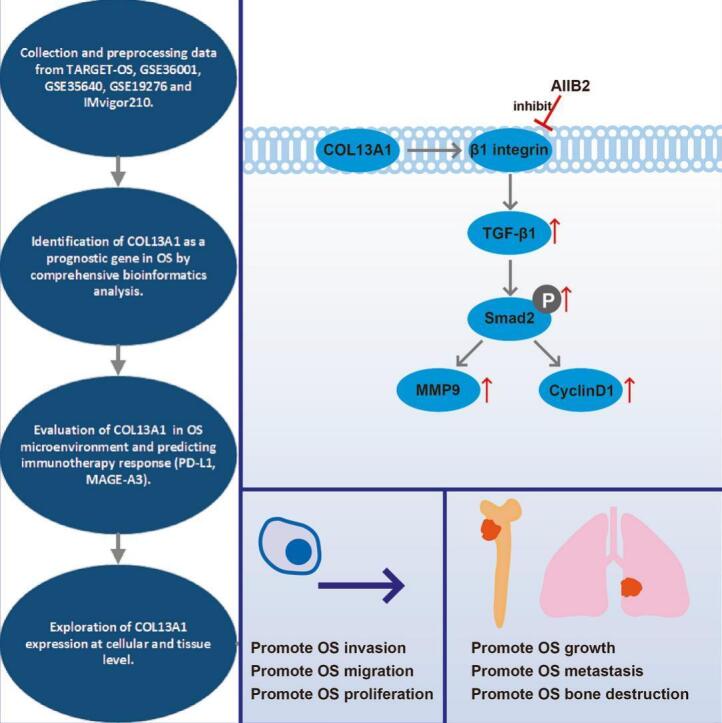

## 1 Introduction

With early metastasis and poor prognosis, OS has received scientists' attention in the last several decades [1]. In the 1980s, the main treatment of OS was surgery resection combined with chemotherapy, which lowered the risk of reocurrence and metastasis [2]. Although this treatment did improve the prognosis of 60 % of OS patients, major breakthroughs in the treatment for OS have been limited since then. What’s worse, nearly 20 % of patients have developed metastases when they are diagnosed with OS [3]. Therefore, exploring new targets and implementing individualized treatment are of great significance to improve the outcome of OS.

Collagen is widely distributed in the extracellular matrix, accounting for about 1/3 of the total protein [4]. Recent studies have revealed that many collagens participate in tumor progression. Type IV collagen in the stroma of extrahepatic bile duct carcinomas could promote tumor progression [5]. Previous research showed that reduced collagen XVIII was significantly related to the cutaneous squamous cell carcinoma progression [6]. Moreover, COL11A1 was found to accelerate Neuroblastoma invasion in Truong’s study [7]. Another type XXIII collagen was detected to be highly expressed in human prostate cancer and could be used as an independent risk factor like prostate-specific antigen [8]. Collagen XIII, encoded by COL13A1, is also a member of the collagen family. However, its role in cancer pathogenesis remains less explored. A previous study revealed that collagen XIII could accelerate breast cancer metastasis [9]. Another research showed that high expression of COL13A1 could lead to bladder cancer recurrence [10]. While COL13A1 has been implicated in OS prognosis through bioinformatics analyses, our study provides the experimental validation of its functional role in driving OS progression via TGF-β/Smad2 signaling.

In this study, we identified COL13A1 as an important gene for the diagnosis and prognosis of OS using bioinformatics tools. Besides, COL13A1 could well predict the immunotherapy response and bosutinib and axitinib were identified as potential treatments for OS. Further cellular experiments showed that COL3A1 could regulate the proliferation and invasion of OS cells. Importantly, COL13A1 enhanced TGF-β signaling through β1 integrin in OS cells. In addition, low expression of COL13A1 could inhibit the growth of OS, reduce bone destruction and lung metastasis *in vivo*. These results demonstrated that COL13A1 is a prognostic predictor for OS and a potential therapeutic target requiring further investigation.

## 2 Materials and methods

### 2.1 Sample collection and data processing

OS datasets TARGET-OS, GSE19276 and GSE36001 were downloaded from TARGET and Gene Expression Omnibus (GEO) database. OS datasets GSE36001 [11] including 19 OS cells and 6 normal bone samples while GSE19276 dataset consisted of 5 normal bone samples and 44 OS samples. Lowess normalization was applied in GSE19276 [12] and quantile normalization was performed in GSE36001. Samples without full clinical characteristics were excluded from this study.

Immunotherapy data sets GSE35640 [13] and IMvigor210 [14] were also collected. IMvigor210 and GSE35640 were normalized by trimmed mean of M-values and RMA, respectively. Three OS samples and the corresponding normal tissue were obtained from department of orthopedics, Zhongnan Hospital of Wuhan University. Detailed information of these datasets was shown in Table S1. The clinical characteristics of high and low COL13A1 expression groups in TARGET-OS were shown in Table S2. The flowchart was shown in Fig.S1.

### 2.2 Weighted gene co-expression network analysis

The overlapped genes in transcriptome of TARGET-OS, GSE36001 and GSE19276 data sets were used for WGCNA by using WGCNA package [15] in R software. At least 30 genes made up a module. Similar modules were merged into one module when cutoff < 0.25. Finally, different gene modules were obtained for subsequent study. Principal component analysis (PCA) was performed for all genes in each module, and the first principal component was regarded as Epigengene. The correlation between gene and clinical information was calculated, and the association between significant module and prognosis of OS was analyzed. Here we collected epigengene of each module and performed univariate Cox regression analysis to further screen the prognostic modules in OS.

### 2.3 Gene set enrichment analysis

TARGET-OS was divided into high and low groups according to the expression of COL13A1. Significant pathways between these two groups were identified using GSEA [16]. False discovery rate (FDR) < 0.05 and *p* < 0.05 were considered significant.

### 2.4 Cell lines and cell transfection

The human OS cell lines 143B, MG63, U2OS, and HOS were cultured at 37 °C. In contrast, the human osteoblast cell line hFOB 1.19 was cultured at 34 °C in a 5 % CO2 incubator (Thermo Fisher, USA). The 143B cell lines were maintained in RPMI-1640 (Hyclone, USA), U2OS cell lines were cultured in McCoy’s 5A medium (Hyclone, USA), and hFOB 1.19 cell lines were grown in DMEM (Hyclone, USA). MNNG/HOS cell lines were cultivated in MEM (Hyclone, USA). All cell culture medium were supplemented with 10 % fetal bovine serum (FBS) and 1 % penicillin–streptomycin.

The siRNA targeting COL13A1 (Ruibo Bio, Suzhou, China; Cat# RB-siCOL13A1) was transfected at 50 nM into osteosarcoma cell lines (143B and HOS) using Lipofectamine 3000 (Invitrogen). Cells were seeded at 5 × 10^4^ cells/well in 6-well plates with DMEM supplemented with 10 % FBS and 1 % penicillin–streptomycin 24 h prior to transfection. After 48 h of treatment, cells were harvested for analysis. For lentiviral knockdown, COL13A1-targeting lentivirus (HanBio, Wuhan, China; Cat# HB-LV-shCOL13A1) was used to infect cells at an MOI of 20. Cells were seeded at 3 × 10^4^ cells/well in RPMI-1640 with 10 % FBS, followed by 72-hour puromycin selection (5 μg/mL). For overexpression, the COL13A1 ORF (cloned into pCDH-CMV-MCS-EF1-Puro; sequence in Table S4) was co-transfected with packaging plasmids pSPAX2 and pMD2.G into 293 T cells (seeded at 2 × 10^6^ cells/10-cm dish in DMEM/10 % FBS). Viral supernatant was collected after 72 h, concentrated via ultracentrifugation, and used to infect OS cell lines. Stable pools were selected with 5 μg/mL puromycin for 72 h. All plasmid and lentiviral sequences are provided in Table S4.

### 2.5 RT-qPCR

Total RNA was extracted from osteosarcoma cell lines (143B and HOS) using TRIzol reagent (Invitrogen Cat# 15596026), with RNA concentration measured via NanoDrop 2000 (Thermo Fisher). The cDNA synthesis was performed using 1 μg RNA with a reverse transcriptase kit (Vazyme, China). The qPCR amplification used SYBR qPCR Master Mix (Vazyme, China). Data analysis employed the 2−ΔΔCt method normalized to GAPDH, the primers used for RT–qPCR are listed in Table S3.

### 2.6 Western blot

Cells were lysed in RIPA buffer (Beyotime, China, P0013B) containing protease inhibitors on ice. After centrifugation (13,000 rpm, 4 °C, 20 min), protein concentration was determined by BCA assay (Biosharp, China, BL521B). Total protein (30 µg per lane) was separated by SDS-PAGE. Membranes were blocked with 5 % skim milk (Biofroxx, CAS: 68514-61-4) in TBST for 2 h at room temperature, then incubated with primary antibodies overnight at 4 °C. The following antibodies were used: COL13A1 (Rabbit polyclonal, Affinity, Cat# AF0598, 1:500), Smad2/3 (Rabbit polyclonal, Servicebio, GB111844, 1:500), p-Samd2 (Rabbit polyclonal, Servicebio, GB13196-2, 1:500), TGF-β1 (Rabbit polyclonal, Affinity, Cat# AF1027, 1:500), Cyclin D1 (Mouse monoclonal, proteintech, Cat No. 60186–1-Ig, 1:500), MMP9 (Rabbit polyclonal, proteintech, Cat No. 10375–2-AP, 1:500), alpha tubulin (Rabbit polyclonal, proteintech, Cat No. 11224–1-AP, 1:500). Then, the membranes were washed with TBST buffer three times and incubated with the corresponding HRP-conjugated secondary antibody at room temperature for 2 h.

### 2.7 Wound healing assay

OS cells (143B or HOS cell lines) were seeded at 5 × 10^5^ cells/well in 6-well plates. At 90 % confluency, a scratch was generated using a sterile 200 μL pipette tip. After three PBS washes, cells were maintained in low-serum medium (DMEM + 1 % FBS) and incubated at 37 °C. Phase-contrast images (0 h, 24 h) were acquired with an inverted microscope (Nikon, Japan).

### 2.8 Cell invasion assay

Cell invasion assays used 8 μm pore transwell chambers pre-coated with Matrigel (Hanbio). 143B or HOS cell lines (5 × 104 cells in 100 μL serum-free DMEM) were seeded in upper chambers, with lower chambers containing 600 μL complete medium (DMEM + 10 % FBS). After 24 h at 37 °C, non-invaded cells were removed by PBS washing. Cells invading to the membrane's lower surface were fixed with 4 % formaldehyde (30 min), stained with 0.5 % crystal violet (30 min), and counted in five random microscopic fields.

### 2.9 Colony formation assay

The 1 × 10^3^ 143B or HOS cell lines were seeded per well in six-well plates and cultured in complete medium (DMEM + 10 % FBS) for 10 days at 37 °C. Colonies were fixed with methanol (30 min, RT), stained with 0.1 % crystal violet (15 min, RT), and colonies (>50 cells) counted microscopically.

### 2.10 Tumor xenograft model

The orthotopic xenograft mouse model was constructed using four-week-old male nude mice injected with 4*10^6^ 143B cells and randomly divided into two groups: Sh-COL13A1 and negative control (NC). Body weight of tumor-bearing nude mice was recorded every other day. After 9 days, tumors were collected by surgery. All animal experiments were approved by the institutional ethics board of Zhongnan hospital of Wuhan University.

### 2.11 Micro-CT analysis

The tibias of mice were collected for micro-CT analysis using skyScan 1176 high-resolution micro-CT imaging system (Bruker, Germany). The micro-CT scanning parameters were as follows: Voltage 50 kV, current 200 μA, scanning time 15 min, size of aluminium flter 0.25 mm, isometric resolution 7 μm. NRecon (Bruker, Germany) and CTAn (Bruker, Germany) were used to reconstruct and quantify the results of micro-CT.

### 2.12 Immunohistochemistry and immunofluorescence staining

IHC was performed on 4 μm paraffin-embedded sections to evaluate COL13A1 expression (Affinity, Cat#AF0598; 1:200). For IF, sections were equilibrated (30 min, RT), PBS-washed, blocked with 5 % BSA/PBS (1 h, RT), then incubated with primary antibody (4 °C, overnight). After PBS washes, fluorescent secondary antibodies were applied (2 h, RT). Images were acquired using a Leica SP8 confocal microscope and analyzed with LAS X.

### 2.13 Statistical analysis

Graph pad prism 5, Statistical Product and Service Solutions software (SPSS 22.0) and R 3.6.2 were used for data analysis. Cox regression analysis [17] was used to explore prognostic modules in TARGET-OS. Log-rank test, survival package and survminer package [18] were used for survival analysis and setting cut-off point. FactoMineR package [19] was used for PCA. The immune infiltration of TARGET-OS was analyzed by CIBERSORT [20]. The pRRophetic package [21] was used to predict targeted drugs for the treatment of OS. The ROC curve was conducted by timeROC package [22] and pROC package [23], respectively. All data was presented in mean ± s.e.m.. Student’s *t*-test or one-way ANOVA were used to analyze the data and *p* < 0.05 was considered significant.

## 3 Results

### 3.1 Integrative WGCNA analysis identified COL13A1 as a prognostic gene in OS

**Fig. 1:**
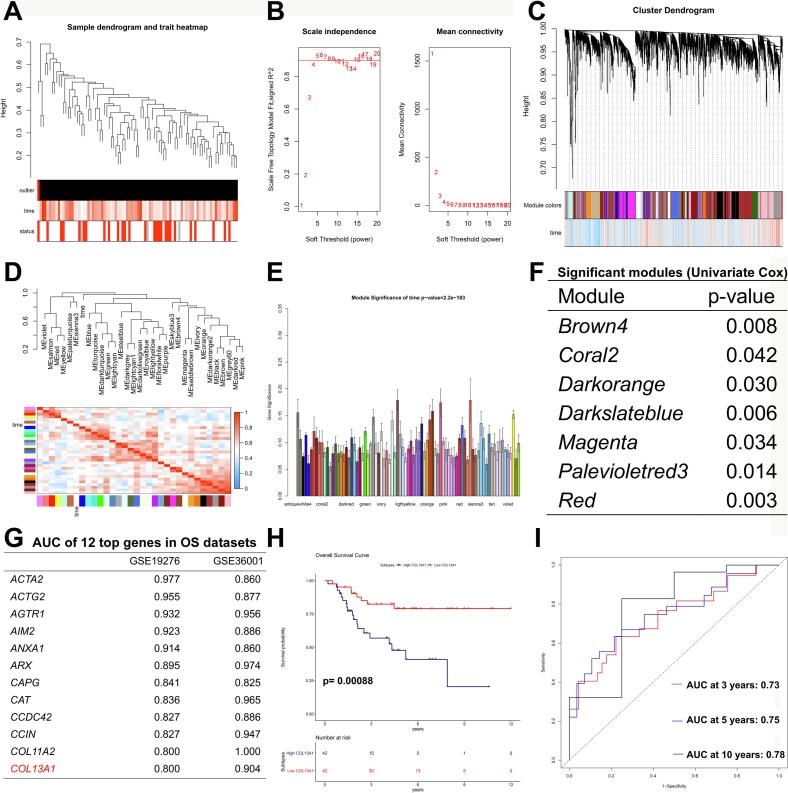
Identification of COL13A1 as a prognostic gene in OS. (A) Cluster dendrogram analysis in TARGET-OS (n = 88). (B) Selection of power value. (C) WGCNA cluster dendrogram and module assignment. The branches refer to clusters of genes that are highly connected. The colors in the horizontal bar represent the modules. (D) Heatmap of module eigenvalue. (E) Gene significances of each module. (F) The result of univariate Cox regression analysis screening prognostic modules. (G) The genes with higher AUC diagnosing OS (GSE19276: n = 49, GSE36001: n = 25). (H) The survival analysis of COL13A1. (I) The AUC of COL13A1 predicting the 3, 5 and 10-year survival of OS. Cox regression analysis was used to explore prognostic modules in TARGET-OS.

Following gene set intersection across TARGET-OS, GSE19276, and GSE36001 cohorts, we performed weighted gene co-expression network analysis (WGCNA) on TARGET-OS (Fig. 1A-E). Here we chose 4 as the power value and scale free R^2^ was 0.86 (Fig. 1A-B) and these genes were divided into multiple modules by dynamic tree cut (Fig. 1C). Each module contained a different number of genes and Fig. 1D-E showed the heat map of module eigenvalue and gene significance of time among different modules, respectively. Moreover, epigengene of each module were used for univariate Cox regression analysis to screen significant modules correlated with prognosis of OS. The results showed that 7 modules including Brown4 module (p = 0.008), Coral2 module (p = 0.042), Darkorange module (p = 0.030), Darkslateblue module (p = 0.006), Magenta module (p = 0.034), Palevioletred3 module (p = 0.014) and Red module (p = 0.003) were significantly correlated with the prognosis of OS (Fig. 1F). These 7 modules including 1319 genes were used for subsequent study. Next, we explored the role of these genes in diagnosis of OS in GSE19276 and GSE36001 and genes with higher area under curve (AUC) were shown in Fig. 1G. Among them, COL13A1, a gene that has hardly been studied in OS attracted our attention. We further explored its prognostic value through survival analysis and the result was shown in Fig. 1H. High COL13A1 expression group showed a significantly poorer prognosis than low COL13A1 expression group (*p* = 0.001). In addition, the AUC of COL13A1 for predicting the 3, 5 and 10-years-survival of OS were 0.73, 0.75 and 0.78, respectively (Fig. 1I). The above results suggested that COL13A1 could be closely related to the prognosis of OS.

### 3.2 COL13A1 was associated with inhibition of M1 macrophage polarization and predicted immunotherapy response

**Fig. 2:**
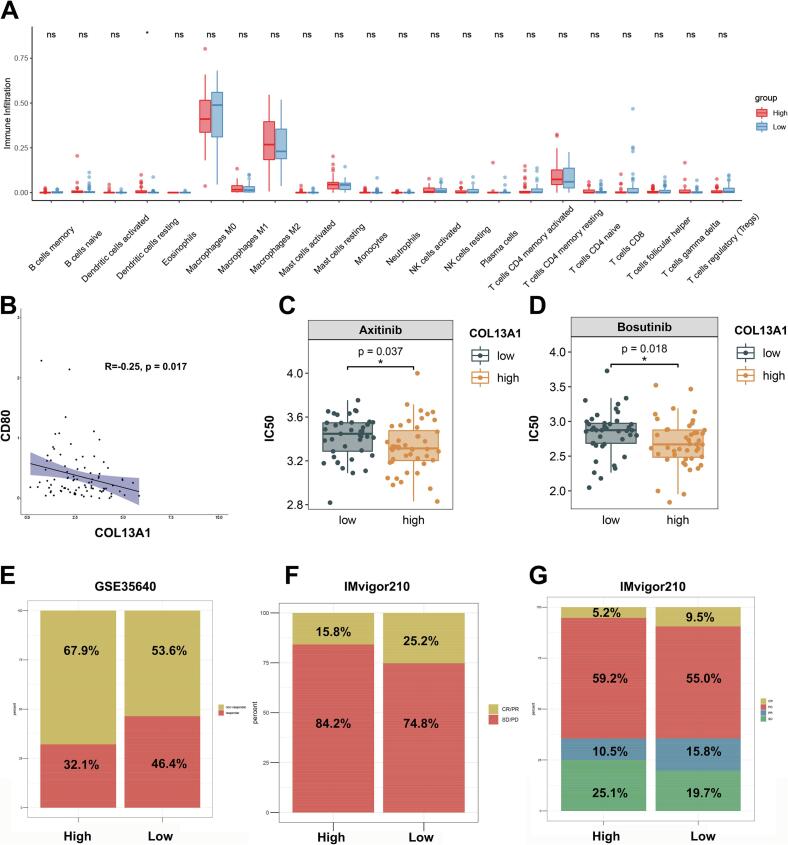
COL13A1 was associated with inhibition of M1 macrophage polarization and predicted immunotherapy response. (A) The proportion of immune cells between high COL13A1 and low COL13A1 expression groups in TARGET-OS (n = 88). (B) The correlation plot between COL13A1 and CD80 (n = 88). (C)-(D) The IC50 of axitinib and bosutinib between high COL13A1 and low COL13A1 expression groups (n = 88). (E) The proportion of immunotherapy response between high COL13A1 and low COL13A1 expression groups in GSE35640 (n = 65). (F)-(G) The proportion of immunotherapy response between high COL13A1 and low COL13A1 expression groups in IMvigor210 (n = 348). Student’s *t*-test or one-way ANOVA were used to analyze the data and p < 0.05 was considered significant. p < 0.05*, p < 0.01**, p < 0.001***, p < 0.0001****, ns, no significance.

Considering the prognostic value of COL13A1 in OS, we further explored its role in the OS microenvironment. The immune infiltration of OS among low and high COL13A1 expression groups was shown in Fig. 2A. The proportion of M2 macrophage in high COL13A1 expression group was higher than that in low COL13A1 expression group. We also explored the relationship between COL13A1 and M1 macrophage marker CD80 (Fig. 2B). The result showed that CD80 was negatively correlated with the expression of COL13A1 (R = −0.25, *p* = 0.017). In addition, the potential treatment for OS suggested that axinitib (p = 0.037) and bosutinib (p = 0.018) may exhibit lower IC50 values in the high COL13A1 expression group (Fig. 2C, D). We also explored the role of COL13A1 in predicting the response of immunotherapy datasets. In Fig. 2E, compared with low COL13A1 expression group (46.4 %), the proportion of responder to MAGE-A3 therapy in high COL13A1 expression group was lower (32.1 %), indicating higher expression of COL13A1 might be correlated with poor immunotherapy response. A similar trend was also seen in IMvigor210 data set that the proportion of responder to PD-L1 in high COL13A1 expression group (15.8 %) was lower than that in low COL13A1 expression group (25.2 %)(Fig. 2F-G).

### 3.3 COL13A1 was highly expressed in OS

**Fig. 3:**
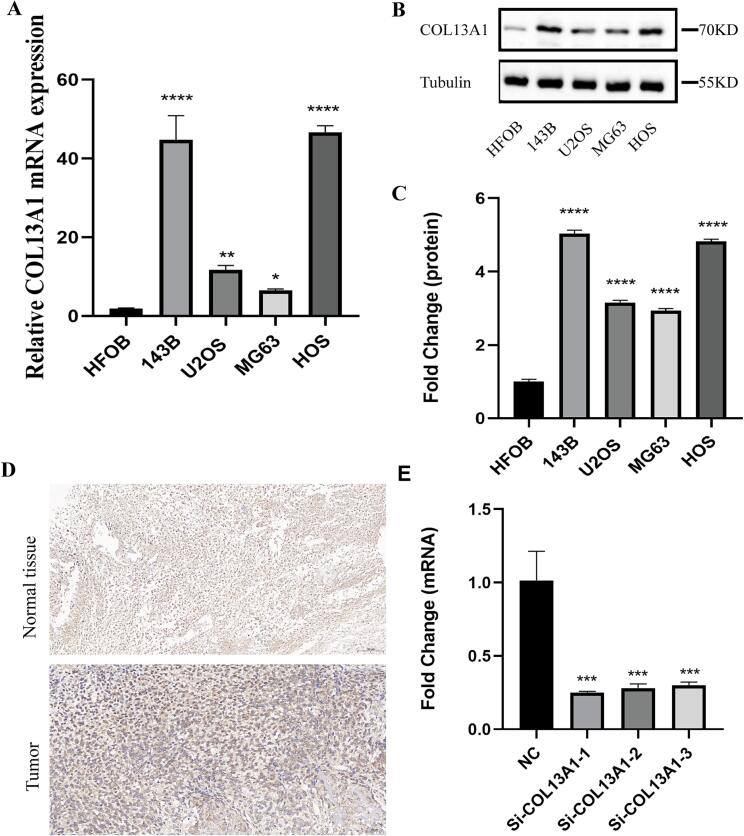
COL13A1 was highly expressed in OS cell lines and tissues. (A) The mRNA expression of COL13A1 in hfOB, 143B, HOS, U2OS and MG63 (n = 3). (B) –(C) The result of western blot of COL13A1 in hfOB, 143B, HOS, U2OS and MG63 (n = 3). (D) The immunohistochemistry of COL13A1 in normal tissue and OS tissue. (E) The knockdown efficiency of three siRNAs against COL13A1 (n = 3). Student’s *t*-test or one-way ANOVA were used to analyze the data and p < 0.05 was considered significant. p < 0.05*, p < 0.01**, p < 0.001***, p < 0.0001****, ns, no significance.

Since COL13A1 had a great impact on the OS progression and well predicted immunotherapy response, we further explored the expression of COL13A1 in OS through RT-qPCR and western blot. Compared with hfOB, the mRNA expression of COL13A1 in 143B (*p* < 0.0001), U2OS (*p* < 0.01), MG63 (*p* < 0.05) and HOS (*p* < 0.0001) were significantly higher (Fig. 3A). COL13A1 protein expression was confirmed by western blot across all cell lines (Fig. 3B-C). Besides, immunohistochemical analysis of clinical samples further revealed that the expression of COL13A1 were higher in OS tissue compared with that in normal tissue (Fig. 3D). In addition, siRNA-mediated silencing achieved efficient COL13A1 knockdown (Fig. 3E), with si-COL13A1-1 selected for lentiviral packaging due to superior efficacy (knockdown efficiency greater than 70 %). These results substantiate bioinformatic findings, which further indicated that COL13A1 could be involved in OS progression.

### 3.4 COL13A1 deficiency represses OS proliferation, migration and invasion

**Fig. 4:**
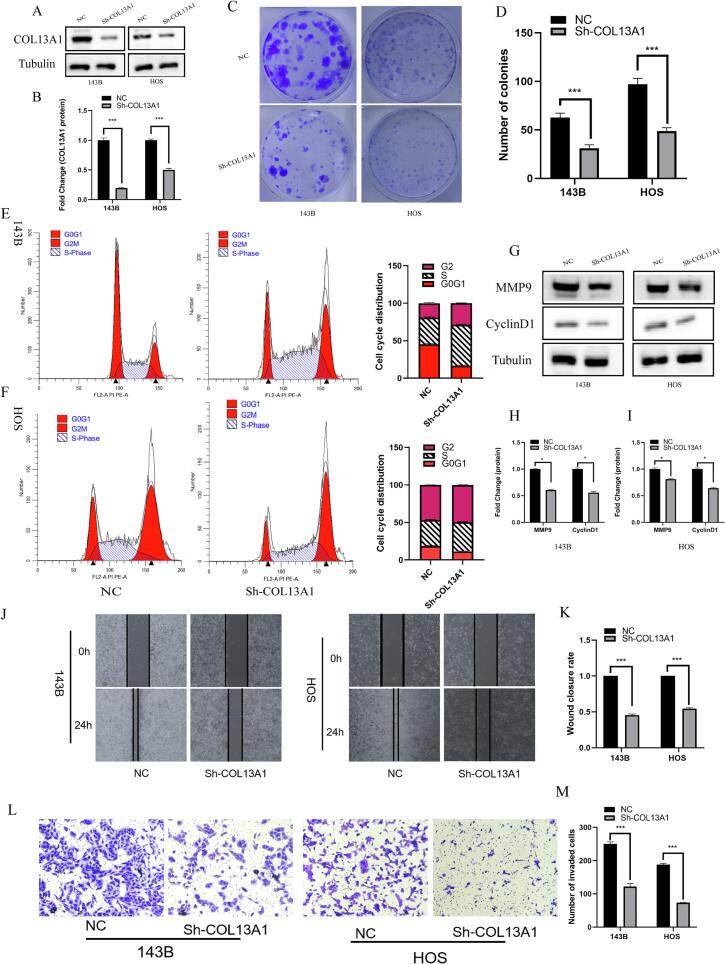
COL13A1 knockdown inhibited OS cell invasion and proliferation. (A)-(B) The result of western blot showing COL13A1 knockdown (n = 3). (C)-(D) The colony formation assay between Sh-COL13A1 and negative control in 143B and HOS cell lines (n = 3). (E)-(F) The cell cycle assay between sh-COL13A1 and negative control in 143B and HOS cell lines (n = 3). (G-I) The result of western blot of MMP9 and CyclinD1 following transfection (n = 3). (J)-(K) The wound healing assay between sh-COL13A1 and negative control in 143B and HOS cell lines (n = 3).(L)-(M) The transwell assay between sh-COL13A1 and negative control in 143B and HOS cell lines (n = 3). Student’s *t*-test or one-way ANOVA were used to analyze the data and p < 0.05 was considered significant. p < 0.05*, p < 0.01**, p < 0.001***, p < 0.0001****, ns, no significance.

Considering huge expression difference of COL13A1 between hfOB and 143B/HOS cell lines, these OS cell lines were selected for functional characterization. We further explored the function of COL13A1 in OS cells. After silencing the expression of COL13A1 in 143B and HOS cell lines through lentivirus (Fig. 4A-B), the results of colony formation experiment showed that the number of colonies was reduced significantly in both OS cells (*p* < 0.001, Fig. 4C-D). Besides, the results of cell cycle also indicated that low expression of COL13A1 could prolong S phase of OS cells and further inhibited cell proliferation (Fig. 4E-F). The expression of MMP9 and cyclinD1 protein were also detected and the result were shown in Fig. 4G-I. The expression of MMP9 and Cyclin D1 decreased significantly in both OS cells after silencing COL13A1 (*p* < 0.05). In addition, the results of wound healing assay showed that 143B cells with COL13A1 knockdown displayed a significant delayed migration compared with the NC (*p* < 0.001) after 24 h and a similar trend was also seen in HOS cells (Fig. 4J-K, p < 0.001). We also explored the role of COL13A1 in the invasion of OS cells and the results were shown in Fig. 4L-M. The number of invasion cells decreased significantly in two OS cells with COL13A1 knockdown (p < 0.001). This finding indicates that the COL13A1 deficiency represses OS proliferation, migration and invasion.

### 3.5 COL13A1 overexpression promotes OS proliferation, migration and invasion

**Fig. 5:**
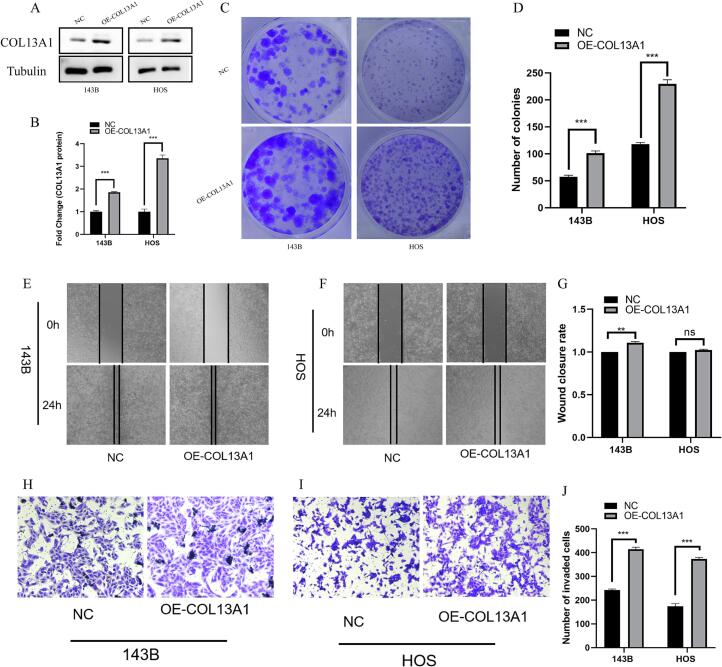
COL13A1 overexpression promoted OS cell invasion and proliferation. (A)-(B) The result of western blot showing overexpressed COL13A1 (n = 3). (C)-(D) The colony formation assay between OE-COL13A1 and negative control in 143B and HOS cell lines (n = 3). (E)-(G) The wound healing assay between OE-COL13A1 and negative control in 143B and HOS cell lines (n = 3). (H)-(J) The transwell assay between OE-COL13A1 and negative control in 143B and HOS cell lines (n = 3). Student’s *t*-test or one-way ANOVA were used to analyze the data and p < 0.05 was considered significant. p < 0.05*, p < 0.01**, p < 0.001***, p < 0.0001****, ns, no significance.

Subsequently, we explored the role of overexpressed COL13A1 in OS cell lines 143B and HOS. In Fig. 5A-B, the results of western blot verified that COL13A1 was significantly overexpressed in 143B and HOS cell lines (*p* < 0.001). The result of colony formation assay also indicated that COL13A1 overexpression could lead to increased number of colonies in OS cells (Fig. 5C-D). Besides, 143B cell lines with overexpressed COL13A1 exhibited significantly higher wound closure rate (*p* < 0.01) while in HOS cell lines no significant change was observed among cells with overexpressed COL13A1 and the NC group (Fig. 5E-G). Then the transwell assay was performed and the results revealed that overexpressed COL13A1 could enhance the invasion of OS cells (Fig. 5H-J). The above results revealed that COL13A1 could promote OS cells invasion, migration and proliferation.

### 3.6 COL13A1 enhanced TGF-β signaling through β1 integrin in OS cells

**Fig. 6:**
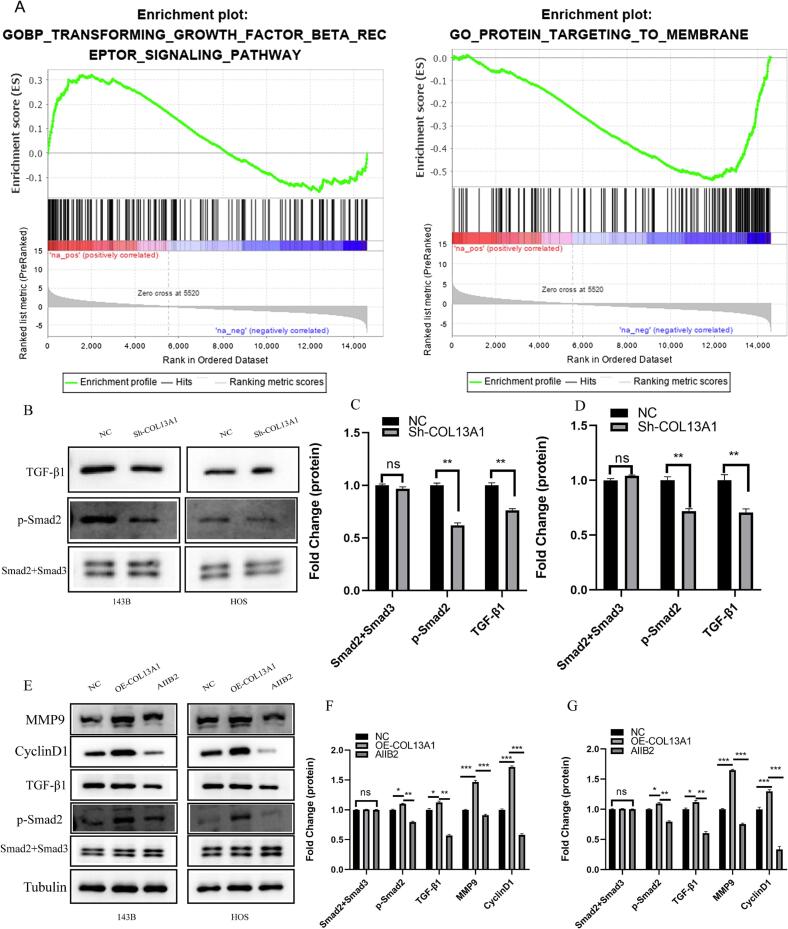
COL13A1 enhanced TGF-β signaling through β1 integrin. (A) The prediction of potential mechanism of COL13A1 regulating OS progression by GSEA. (B)-(D) The western blot of 143B and HOS cells with COL13A1 knockdown (n = 3). (E)-(G) The western blot analysis of 143B and HOS cells with overexpressed COL13A1 and AIIB2 (n = 3). Student’s *t*-test or one-way ANOVA were used to analyze the data and p < 0.05 was considered significant. p < 0.05*, p < 0.01**, p < 0.001***, p < 0.0001****, ns, no significance.

Next, the underlying mechanism of COL13A1 affecting the progression of OS was explored. The result of GSEA was shown in Fig. 6A and Table S5-6. High COL13A1 expression was correlated with TGF-β signaling while low COL13A1 expression was related to protein targeting to membrane. The above bioinformatics analysis showed that TGF-β signaling might be an important pathway for COL13A1 to affect the prognosis of OS. Therefore, further experiments were conducted to verify the results of GSEA. In Fig. 6B-D, after silencing COL13A1, the protein level of TGF-β1 and p-Smad2 decreased significantly (*p* < 0.05) in 143B and HOS cell lines. β1 integrin was considered to regulate TGF-β signaling [24]. Therefore, we further explored whether COL13A1 regulated TGF-β pathway through β1 integrin, thereby promoting the progress of OS. As shown in Fig. 6E-G, overexpressed COL13A1 significantly increased the expression of MMP9, Cyclin D1, p-Smad2 and TGF-β1. However, after adding the β1 integrin blocker AIIB2, the effect of COL13A1 enhancing TGF-β signaling was inhibited in both OS cell lines, which further indicated that COL13A1 regulated TGF-β signaling through β1 integrin. Together, these findings demonstrate that COL13A1 functionally activates TGF-β signaling through β1 integrin, further unregulated the expression of MMP9 and Cyclin D1.

### 3.7 COL13A1 deficiency inhibited OS growth and metastasis and reduced bone destruction *in vivo*

**Fig. 7:**
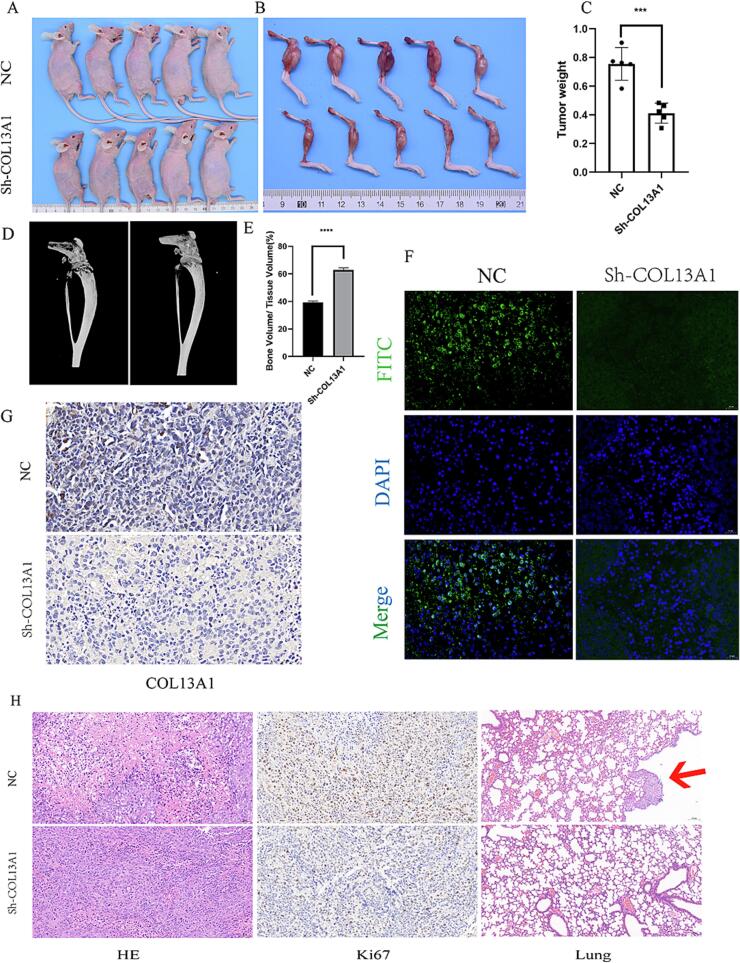
The role of COL13A1 in OS *in vivo*. (A)-(C) The volume and weight of tumor in nude mice between sh-COL13A1 and negative control (n = 5). (D)-(E) The result of micro-CT of bone mass (n = 5). (F)-(G) The immunohistochemistry and immunofluorescence staining of specimen from nude mice between sh-COL13A1 and negative control. (H) The HE, ki67 and lung tissue staining between sh-COL13A1 and negative control. Student’s *t*-test or one-way ANOVA were used to analyze the data and p < 0.05 was considered significant. p < 0.05*, p < 0.01**, p < 0.001***, p < 0.0001****, ns, no significance.

Next, we constructed the orthotopic xenograft mouse model to further reveal the role of COL13A1 in the progression and metastasis of OS and its effect on bone destruction *in vivo*. NC and COL13A1-knockdown 143B cells were injected to right tibia of the nude mice (using 5 mice per group). Nine days after the injection, the mice were sacrificed and the weight of the OS was calculated and the results were shown in Fig. 7A-C. The weight of OS in sh-COL13A1 group were lower than NC group (*p* < 0.05). The result of micro-CT also showed that silencing COL13A1 could reduce the bone destruction in mice (*p* < 0.0001, Fig. 7D-E). Besides, the results of immunohistochemistry and immunofluorescence also indicated the COL13A1 deficiency in OS sections of nude mice compared with the NC group (Fig. 7F-G). Finally, in Fig. 7H, the HE and Ki67 staining indicated that COL13A1 knockdown could inhibit OS growth and the staining from lung of mice also suggested that COL13A1 knockdown could reduce OS lung metastasis. The above results showed that COL13A1 might promote OS progression, lung metastasis and bone destruction.

## 4 Discussion

With a high degree of malignancy and invasion, OS is prone to metastasize in the lung and brain. The survival rate of patients with primary OS after surgical resection is about 65 %, but it will be reduced to less than 25 % if metastasis occurs [25]. Hence, finding novel targets for OS is of great significance. The survival analysis indicated that high expression of COL13A1 was found to be related to the poor prognosis of OS. A recent research also indicated that COL13A1 contributed to the relapse of the seminoma [26], which implied that COL13A1 might be an important biomarker in multiple tumors. However, these studies mainly focused on the bioinformatics analysis from public database and lacked further experimental exploration. We also reported that high expression of COL13A1 was correlated with the inhibition of M1 macrophage. In Solomon’s study M1 macrophage could improve the prognosis of mice with Ehrlich ascites carcinoma [27]. Besides, we identified axitinib and bosutinib as potential treatment for OS. Axitinib has been widely used in immunotherapy for lung cancer [28] and thyroid cancer [29]. Bosutinib was also found to treat chronic myelogenous leukemia [30] and Head and neck squamous cell carcinoma [31]. Therefore, these two drugs might be also promising treatment for OS. Besides, high expression of COL13A1 was related to the poorer immunotherapy response, indicating that COL13A1 might regulate tumor immunotherapy. The results of transwell, colony formation and wound healing assay showed that COL13A1 promoted OS cells migration and proliferation. A previous study showed that COL13A1 was upregulated in OS patients through bioinformatics analysis [32]. Another study also showed that COL13A1 was involved in immune status and DNA methylation of OS through multi-omics analysis [33]. These literature reports were consistent with the result of our analysis, which also suggested that COL13A1 might be important for OS progression.

TGF-β signaling has been studied and related to tumor progression[34]. A recent study showed that TGF-β could induce metastasis of endometrial carcinoma [35]. Another research also revealed that TGF-β promoted the polarization of M2 macrophage and accelerated the tumor growth in prostate cancer [36]. In addition, TGF-β1 was found to be upregulated by IGFBP7 in esophageal squamous cell carcinoma [37]. A previous research also implied that TGF-β1 suppressed the proliferation of thyroid carcinoma through ERK1 signaling [38]. In this study we performed GSEA and identified TGF-β as an important pathway to regulate OS progression. Further experiments indicated that COL13A1 could enhance the TGF-β signaling. We also reported that high expression of COL13A1 inhibited the expression of MMP9 and Cyclin D1. MMP9 has been proven to promotes tumor invasion and metastasis by degrading extracellular mechanisms and basement membrane. A previous study reported that MMP9 was correlated with invasion and metastasis of squamous cell carcinoma of the uterine cervix [39]. Another study also revealed that MMP9 could be upregulated by ASIC1α in liver cancer[40]. Cyclin D1 is an important regulator of cell cycle, which can bind to cyclin dependent kinases and affect G1/S phases. A previous study showed that cyclinD1 promoted progression of intrahepatic cholangiocarcinoma by inhibiting Dicer expression [41]. A recent study also revealed that decreased expression of Cyclin D1 could inhibit colon cancer cell growth [42]. These reports supported our results and indicated that COL13A1 might promote OS progression through TGF-β signaling.

The animal experiments showed that COL13A1 could promote OS growth, bone destruction and lung metastasis. A recent study showed that COL13A1 was related to bone mineral density in CMS19 disease model [43]. Moreover, in Anne’s research low expression of COL13A1 was correlated with the abnormal bone mass formation [44]. These reports suggested that COL13A1 could also be a regulator of bone mineral density, which provided novel insights in bone metabolism.

While this study demonstrated the role of COL13A1 via TGF-β axis in osteosarcoma, we acknowledge that tumor heterogeneity across histological subtypes may influence its functional outcomes. As highlighted by Jiake Xu and David Wood et al. [45], distinct OS subtypes, including conventional (osteoblastic, chondroblastic, fibroblastic) and other types of OS, exhibit diverse tumor microenvironment (TME) profiles. These subtype-specific TME variations could potentially modulate COL13A1-mediated signaling. Emerging single-cell RNA sequencing evidence indicated that COL13A1 is highly expressed in osteosarcoma and likely promotes tumor progression. COL13A1 showed elevated expression in osteoblastic osteosarcoma cells, cancer-associated fibroblasts (CAFs), and endothelial cells (ECs) [46,47]. Since COL13A1 is predominantly expressed in osteoblastic osteosarcoma cells, this study employed two osteoblastic osteosarcoma cell lines (143B and HOS) to investigate the effects of COL13A1 knockdown or overexpression on osteosarcoma progression.

Although we identified COL13A1 as a prognostic gene in OS, the sample size used for bioinformatics analysis was small and more datasets were needed for further study. At the same time, we also need collect OS clinical samples to better verify our conclusion. Although our study mechanistically delineates COL13A1-driven TGF-β activation via β1 integrin, we acknowledge that the functional rescue through TGF-β pathway inhibition remains unexplored. We will continue to conduct collagen-related research, and we hope more researchers will pay attention to this field. While our findings highlight the role of COL13A1 in OS progression, the therapeutic potential and safety profile of targeting COL13A1 warrant rigorous evaluation in future studies, including comprehensive toxicity assessments.

## 5 Conclusion

Comprehensive bioinformatics tools were used to identify COL13A1 as a prognostic gene and an immunotherapy response predictor in OS. In addition, COL13A1 could promote OS progression, metastasis and bone destruction. We also noticed that COL13A1 enhanced TGF-β signaling through β1 integrin, which suggested that COL13A1/ β1 integrin /TGF-β axis could be a promising target for OS.

## Ethical Approval and Consent to participate

This study was approved by the institutional ethics board of Zhongnan hospital of Wuhan university.

Consent for publication

All the authors have consented for the publication.

## CRediT authorship contribution statement

**Kang-Wen Xiao:** Investigation, Methodology, Visualization, Writing – original draft. **Zhenyi Chen:** Formal analysis, Investigation, Methodology, Writing – original draft. **Chong Zhang:** Data curation, Investigation, Methodology, Validation, Visualization. **Zhiqiang Yang:** Validation, Visualization. **Liangyu Guo:** Validation, Visualization. **Yuanlong Xie:** Investigation, Methodology, Writing – review & editing. **Jun Lei:** Funding acquisition, Writing – review & editing. **Lin Cai:** Funding acquisition, Writing – review & editing.

## Funding

This work was supported by Health Care of Yellow Crane Talent Plan (Project No. 17), Translational Medicine and Interdisciplinary Research Joint Fund of Zhongnan Hospital of Wuhan University (ZNJC201927), Zhongnan Hospital of Wuhan University Medical Science and Technology Innovation Platform Construction Support Project (Grant No. PTXM2021003).

## Declaration of competing interest

The authors declare that they have no known competing financial interests or personal relationships that could have appeared to influence the work reported in this paper.

## Data Availability

The datasets (TARGET-OS, GSE19276, GSE35640 and GSE36001) analyzed during the current study are available in the GEO database (https://www.ncbi.nlm.nih.gov/) and TARGET database (https://ocg.cancer.gov/programs/target). IMvigor210 was collected from a previous study (https://www.nature.com/articles/nature25501).
